# Metformin inhibits the development, and promotes the resensitization, of treatment-resistant breast cancer

**DOI:** 10.1371/journal.pone.0187191

**Published:** 2017-12-06

**Authors:** Gerald Davies, Liubov Lobanova, Wojciech Dawicki, Gary Groot, John R. Gordon, Matthew Bowen, Troy Harkness, Terra Arnason

**Affiliations:** 1 Department of Anatomy and Cell Biology, University of Saskatchewan, Saskatoon, Saskatchewan, Canada; 2 Department of Medicine, University of Saskatchewan, Saskatoon, Saskatchewan, Canada; 3 Department of Surgery, University of Saskatchewan, Saskatoon, Saskatchewan, Canada; University of South Alabama Mitchell Cancer Institute, UNITED STATES

## Abstract

Multiple drug resistant (MDR) malignancy remains a predictable and often terminal event in cancer therapy, and affects individuals with many cancer types, regardless of the stage at which they were originally diagnosed or the interval from last treatment. Protein biomarkers of MDR are not globally used for clinical decision-making, but include the overexpression of drug-efflux pumps (ABC transporter family) such as MDR-1 and BCRP, as well as HIF1α, a stress responsive transcription factor found elevated within many MDR tumors. Here, we present the important *in vitro* discovery that the development of MDR (in breast cancer cells) can be prevented, and that established MDR could be resensitized to therapy, by adjunct treatment with metformin. Metformin is prescribed globally to improve insulin sensitivity, including in those individuals with Type 2 Diabetes Mellitus (DM2). We demonstrate the effectiveness of metformin in resensitizing MDR breast cancer cell lines to their original treatment, and provide evidence that metformin may function through a mechanism involving post-translational histone modifications via an indirect histone deacetylase inhibitor (HDACi) activity. We find that metformin, at low physiological concentrations, reduces the expression of multiple classic protein markers of MDR *in vitro* and in preliminary *in vivo* models. Our demonstration that metformin can prevent MDR development and resensitize MDR cells to chemotherapy *in vitro*, provides important medical relevance towards metformin’s potential clinical use against MDR cancers.

## Introduction

An understanding of how and why tumor cells develop multiple drug resistance (MDR) has remained a significant question in cancer research, and its elucidation may identify future targets to prevent, or reverse, treatment resistance. For many common malignancies, the delayed emergence of MDR following initial successful responses is a devastating event. Breast, colon, lung and hematological cancers are common cancers that have high rates of acquired treatment resistance [1]. Minimally effective therapies, and poorly tolerated side effects from the necessary rescue therapies raises the importance of understanding the underlying biology within this patient population to overcome this important clinical development. Discovering non-toxic, effective treatment options to overcome treatment resistance, or perhaps prevent MDR development in the first place, is of great interest.

Resistance to therapies can be inherent at diagnosis, or acquired in subpopulations of silent surviving cells that reappear years later after an initial apparently successful treatment regime [2]. Resistance to one agent is frequently manifested as resistance to many, hence the term “multiple drug resistance” or MDR. This was recognized many years ago, and is reflected in our current chemotherapy cocktails that incorporate multiple therapeutic agents with unrelated modes of action to decrease the rate of new treatment resistance. Well-recognized molecular mechanisms of resistance are known and have been recently reviewed [3]. At the forefront is the ABC family of drug-efflux pumps whose over-activity has been consistently associated with drug resistance. Despite being novel drug therapy targets, clinical trials using ABC transporter inhibitors have been disappointing in part because of their toxicities [4], but also because they are unlikely to account for all mechanisms of resistance that are present. MDR appears to be a complex and multifactorial adaptation by cancer cells that enables survival of these cell population subsets, and it is clear that individual cancers appear to highjack more than one mechanism, without a single overriding molecular target accountable for all.

Early diagnosis, screening and advances in combination therapy of breast cancer has resulted in longer disease-free survival times and cure rates [5, 6]. Nonetheless, many of these patients return years later with recrudescent tumors [2]. Providing hope for the identification of nontoxic therapies and a means to prevent MDR development in this important population of treatment resistant breast cancer survivors is our ultimate goal. To this end, we primarily utilize breast cancer cell lines selected *in vitro* for treatment resistance as our fundamental model of MDR (see Davies *et al* 2009 and 2014). Below we describe our investigations into the potential utility of metformin as adjunct therapy in the treatment of established MDR and in preventing the development of new treatment resistance. The oral insulin-sensitizing drug metformin is a first line therapeutic in the management of Type 2 diabetes (DM2), and has also been shown to have antiproliferative activity *in vitro* against multiple cancer cells lines [7, 8]. An early meta-analysis performed on DM2 patients taking metformin with cancer reported a 31% reduction in the incidence of new cancers including pancreas, colorectal, breast and lung [9]. Recent meta-analyses confirm that individuals with DM2 who also have lung, colorectal and liver cancer derive significant survival benefits regarding clinical outcomes if also on metformin [10–12]. Patients with breast cancer benefited from metformin treatment in terms of all cause survival, but not in incidence [13]. To date, however, the molecular mechanisms facilitating metformin’s antiproliferative impact remains unclear. It also remains untested whether metformin pretreatment can provide a benefit to established MDR malignancy or interfere with the development of acquired drug resistance.

To study the underlying pathways necessary for the antiproliferative effect of metformin, as well as a direct test of the utility of metformin in preventing acquired drug resistance, we used the widely studied MCF7 breast cancer cell line and selected them for Doxorubicin (DOX) resistance. Our accelerated selection protocol occurs over ~2 weeks, generating cell populations that exhibits enhanced cell viability upon pulse exposure to normally toxic doses of the selected drug, exhibits resistance to previously unexposed drug classes, and expresses high levels of one or more of BCRP, MDR-1, or HIF1α [14]. The following details our studies testing our hypothesis that metformin can potentially reverse and prevent MDR development, and offer a means to elucidate molecular pathways impacted by metformin antiproliferative activity.

## Materials and methods

### Cell culture and methods

MCF7 and T47D ER^+^, and BT-20 and MDA-MB-231 ER^-^ human breast cancer, and K562 leukemia cells were obtained from commercial sources; the American Type Culture Collection (ATCC), USA. The chemicals Doxorubicin hydrochloride (DOX; Pfizer), Tamoxifen (TAM; Cayman Chemical), phenformin (Sigma), Trichostatin A (TSA; Sigma), estradiol (Cayman Chemical), Apicidin (Sigma), and Troglitazone (TRG; Calbiochem) were acquired from the indicated providers. All treatment compounds were reconstituted in dimethylsulfoxide (DMSO) except metformin (Sigma), which was reconstituted in molecular-grade water (Hyclone). The HDACi assay and *in vitro* hypoxia experiments were conducted as previously described [14]. MCF7 and K562 parental cells were selected for drug resistance according to our published methods [14, 15].

### Western blot analysis

Adherent MCF7 cells were scraped, and centrifuged with sterile PBS for collection and resuspended in RIPA buffer followed by pulse sonication. Westerns were performed as described [14]. Antibodies against the following proteins were used, typically at 1:2000 dilution: MDR-1 (Sigma), BCRP (Santa Cruz Biotechnology; SCBt), HIF1α (Abcam), S6K total (SCBt), S6K^S411phos^ (SCBt), p53 (SCBt), p53^S392phos^ (Abcam), TFPI1 (Abcam), AMPKα1/2 total (SCBt), AMPKα1^T183^/2^T172phos^ (Abcam), AKT total (SCBt), AKT^S473phos^ (SCBt), PARP (Sigma), ERα (SCBt), histone H3 total (Millipore), H3^K9Ac^ (Millipore), H2B total (Abcam), H4^K12Ac^ (Abcam), NFκB (SCBt), NREL (SCBt), tubulin (Sigma), actin (Sigma), and GAPDH (Millipore). Luminescence was captured on film (Kodak) with subsequent chemical development. Collection and semiquantitation of Western blots densitometry was done using ImageJ Version 1.51 from scans of the original film.

### MTT and Thymidine incorporation and trypan blue assays of viability and proliferation

MTT (3-(4,5-dimethylthiazol-2-yl)-2,5-diphenyltetrazolium bromide) and a non-radioactive thymidine analogue 5-ethynyl-2’-deoxyuridine (EdU) kit were used to measure cell viability and proliferation. For thymidine, the Click-IT kit (Life Technologies) labeled cells with EdU for 24 hours. For trypan blue assays (TB), MCF7 cells were cultured to 70% confluency in 6-well multiwell tissue culture dishes and treated in triplicate with metformin at the dosages indicated for 48 hours, trypsinized and centrifuged to pellet in microtubes. The pellet was treated with 0.25% TB solution in PBS (Hyclone) and cell viability was assessed using a TC20 automated cell counter (Biorad).

### RNA silencing

MCF7 cells were transfected with 1 μg of duplex human AMPKα1/2, NFκB or scrambled *si*RNA according to the manufacturers’ instructions (Santa Cruz Biotechnology). 1 μg (80 pmol) of targeted *si*RNA was delivered to 50 μl of the cationic delivery agent Lipofectamine RNAiMAX (Gibco) for 30 minutes at RT, then incubated for 72 hours followed by drug treatments.

### Animals

Female NOD/SCID/common gamma-chain knock-out (NSG: NOD/Prkdc^SCID^/IL2RN^-/-^) 8 to 14 week-old mice (Jackson Laboratory, USA) were used, and fed *ad libitum*. All experiments were approved by the University of Saskatchewan animal ethics office, in accord with the guidelines of the Canadian Council on Animal Care.

### Murine xenograft experiments

K562 Doxorubicin-resistant cells (1 x 10^6^ cells/mouse in 100 μL of PBS) were injected subcutaneously into the sacral region. Metformin (130 mg/kg) was administered by intraperitoneal (i.p.) injection on alternating days, starting when tumors were ~25 mm^3^, as measured by calipers every other day (~11 days after seeding tumor cells). Control mice received vehicle only (200 μL PBS). At day 28, tumors were surgically excised and analyzed (n = 2 per treatment arm). The 4–28 tumor was obtained from a patient with triple negative (TN) breast cancer with written informed consent from the donor, and compliant with the Research Ethic board approved protocol at the University of Saskatchewan. A fragment from the original tumor was passaged 8 times in NSG mice; the resultant tumor was excised and ~2 mm fragments were grafted subcutaneously into 4 separate NSG mice. On day 37 metformin was delivered to each mouse as 0, 50, 100 or 200 mg/kg per i.p. injection. The mice were sacrificed after 72 hours of receiving metformin, with tumors surgically removed and analyzed (n = 1 per treatment arm).

### Statistical analysis

Statistical analysis was performed using One-Way ANOVA and Bonferroni correction with Stata 13.0 software. Error bars define the standard error of the mean. Statistically significant differences (p < 0.05) are noted within their respective figure legends.

## Results

### Metformin has antiproliferative potential against drug resistant cell populations *in vitro*, and works additively with Doxorubicin

**Fig 1:**
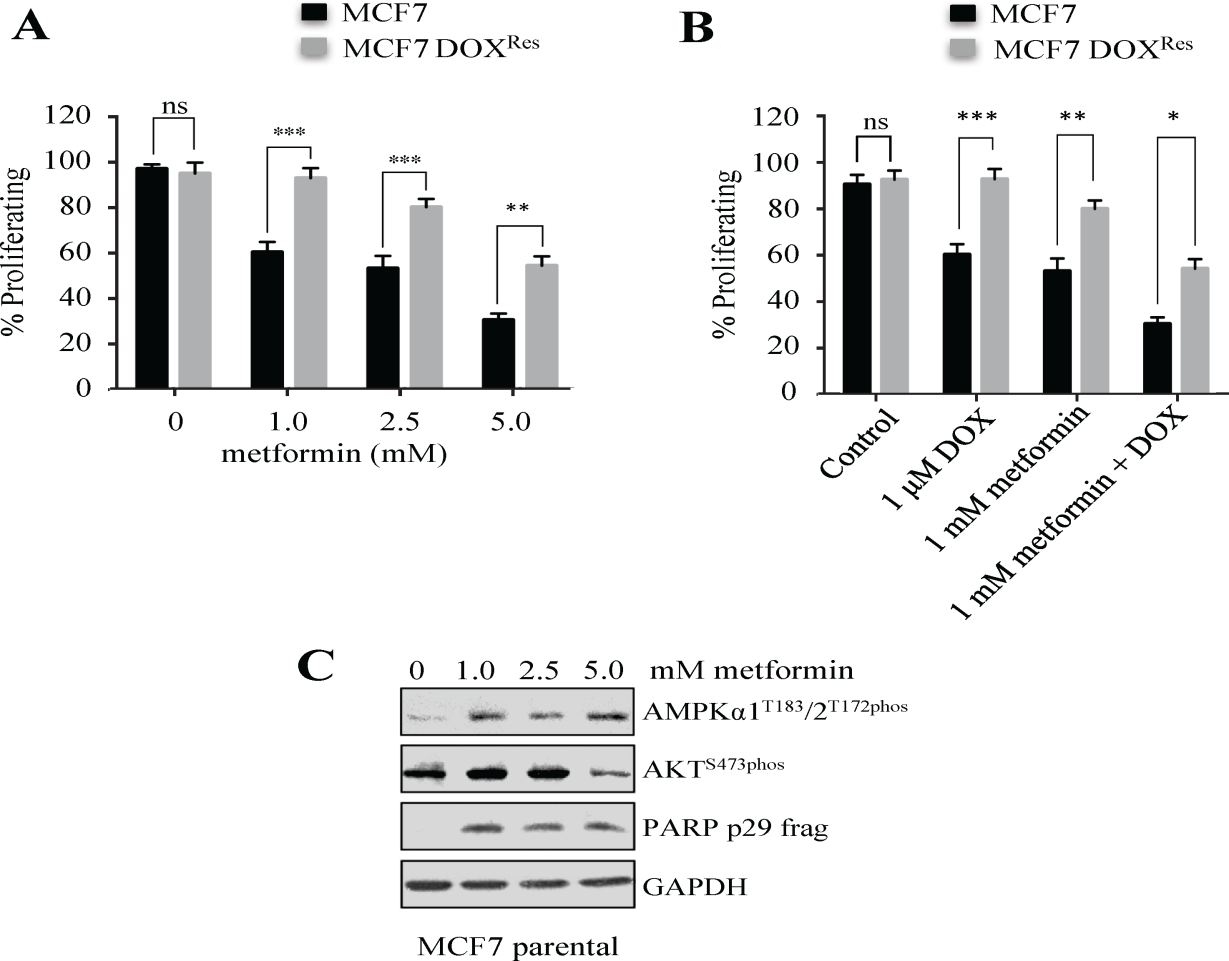
Metformin possesses antiproliferative activity. **(A)** MCF7 parental and DOX^Res^ cells were treated with three doses of metformin over 48 hours, with cell proliferation measured using MTT assays on three biological repeats. **(B)** MCF7 parental and DOX^Res^ cells were treated with metformin and/or DOX for 48 hours, in triplicate, with cell proliferation measured using MTT assays from three biological repeats. **(C)** Protein extracts were prepared from the MCF7 parental cells used in **(A)** and analyzed using Western analysis with the antibodies shown. The blot shown is representative of three reproducible experiments. * = p < 0.05, ** = p < 0.01, *** = p < 0.001, ns = not significant.

Our *in vitro* biological model of drug resistance made use of the well characterized and widely utilized estrogen receptor positive (ERα^+^) MCF7 breast cancer cell line (parental), and its paired cell line selected for Doxorubicin resistance (DOX^Res^), using our previously published *in vitro* protocol [14, 15]. Cells selected against DOX using our protocol are considered MDR due to resistance against unrelated drugs, including metformin [16] (Fig 1A). The dosing of metformin *in vitro* is a contentious issue with concerns that supra-physiological doses result in off-target effects and are not reflective of *in vivo* events. Our *in vitro* metformin dosing ranges were from 0.1 mM to 5 mM; in comparison, the *in vivo* trough human serum levels of metformin (average 1500 mg oral daily dose) has been reported at 2–6 μM [17], with peak levels of 38 μM and steady state ranges of 15.5 μM [18].

We measured the proliferation of treatment sensitive (MCF7 parental) versus resistant (DOX^Res^) MCF7 cell populations following a 48-hour metformin exposure. Metformin impaired growth in a dose-dependent manner in both populations of MCF7, but to a significantly lesser degree in DOX^Res^ cells (Fig 1A). To determine whether metformin could enhance DOX cytotoxicity as combination therapy in MCF7 DOX^Res^ cells, we treated MCF7 parental and DOX^Res^ cells with DOX (1 μM) and metformin (1 mM), individually and in combination. The results show that there is an additive increase in killing when metformin is combined with DOX in both sensitive and resistant cell populations (Fig 1B). Metformin activity was confirmed, as AMPKα1^T183^/2^T172phos^ and AKT^S473phos^ was relatively increased, and decreased, respectively, as expected [19, 20] (Fig 1C). Furthermore, the reduced proliferation observed with metformin exposure was correlated with enhanced apoptosis (Fig 1C) (measured as poly(ADP-ribose) polymerase (PARP) cleavage), as has been previously reported [21, 22].

### Metformin has enhanced antiproliferative potential in ERα^+^ cells and decreases total ERα protein levels

**Fig 2:**
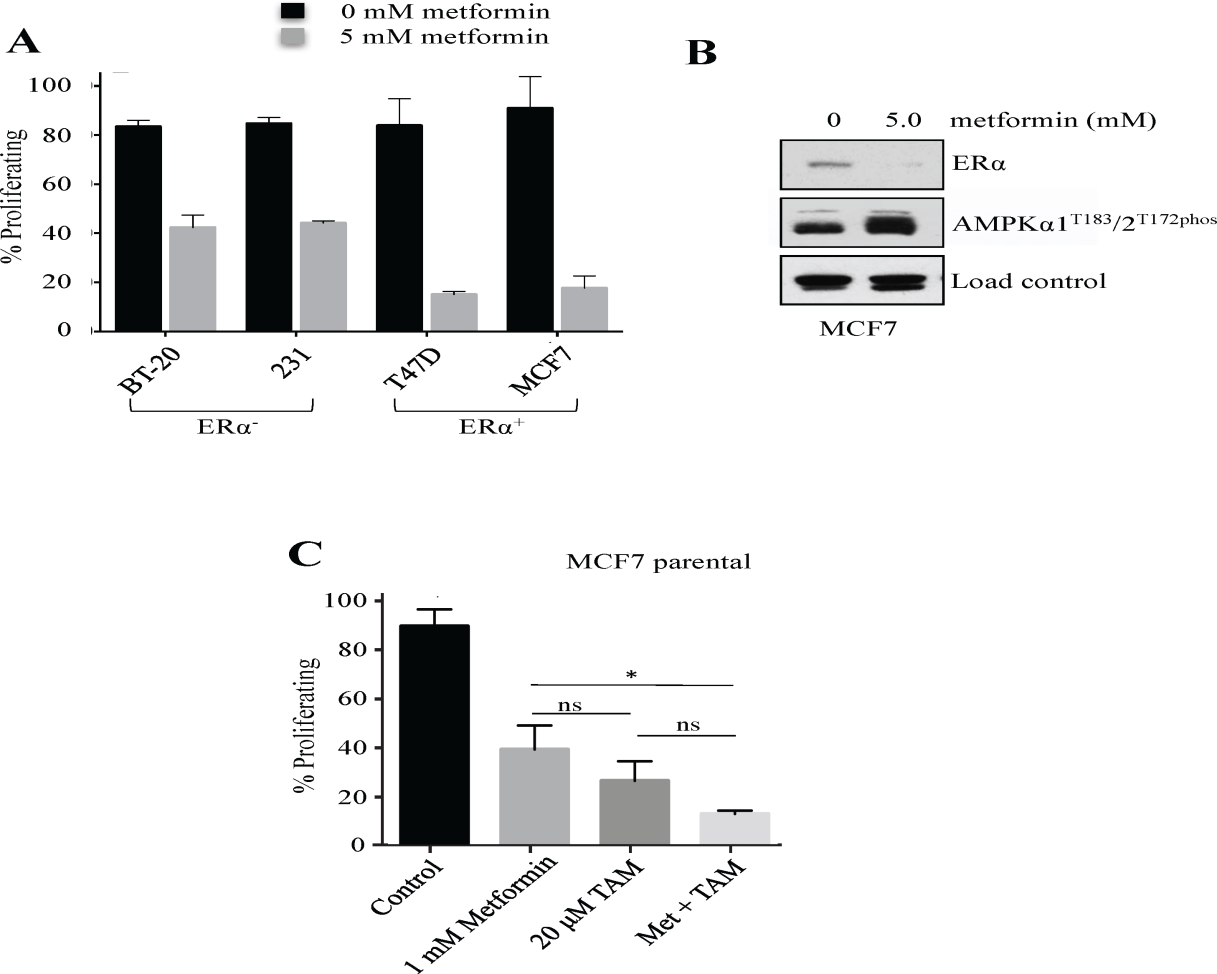
Metformin is effective against ERα^-^ and ERα^+^ breast cancer cells, and decreases ERα levels. **(A)** ERα^−^ (BT-20 and MDA-MB-231) cells, and ERα^+^ (T47D and MCF7) cells were treated, in triplicate, with 5 mM metformin (+), or left untreated (−), for 48 hours, with cell proliferation measured by MTT from two biological repeats. p < 0.001 for all cell lines comparing 0 mM to 5 mM metformin, n = 6 samples analysed. **(B)** Protein lysates were prepared from MCF7 cells treated with 5 mM metformin, or left untreated, for 48 hrs. The lysates were then tested by Western analysis using the antibodies shown. These results are reproducible over three separate experiments. **(C)** MCF7 cells were treated with metformin and/or Tamoxifen (TAM), for 48 hours and analyzed as described above for (A). Values normalized to control. n = 3 samples analyzed. * = p < 0.05.

The estrogen receptor α (ERα^+^) status of breast cancer cells is utilized as both a prognostic and predictive marker of cancer outcomes; triple negative (TN) breast cancers lack the ERα, progesterone (PR) and HER2 receptors and are inherently more difficult to treat, whereas ERα^+^ tumors respond well to therapies and are uniquely suited to adjunct use of anti-estrogen therapy [23]. We showed above that metformin had antiproliferative activity against ERα^+^ cells (Fig 1), and we next investigated whether metformin had a similar, or different, impact on ERα^+^ versus ERα^−^ cell lines. We exposed ERα^+^ (T47D and MCF7) and TN (BT-20 and MD-MBA-231) breast cancer cells to metformin with cell proliferation measured by MTT analyses (Fig 2A). The results show that metformin had antiproliferative potential regardless of ERα status, but had a noticeably greater impact against ERα^+^ cells.

Malignancies of the breast can be hormone-responsive, including acting through estrogen binding to ERα [24], with ER modulators such as tamoxifen typically used as adjuvant therapy [25]. To determine whether metformins’ greater influence on ERα^+^ cells reflects modulation of the ERα protein, we determined ERα total protein levels compared to untreated controls, and noted that 5 mM metformin markedly reduced ERα abundance (Fig 2B). Western analyses of AMPK phosphorylation confirmed the expected AMPK phosphorylation/activation with metformin exposure (Fig 2B). Tamoxifen interferes with ERα function, and we asked whether Tamoxifen and metformin have similar antiproliferative impacts on ERα. We found that the combination of metformin in combination with Tamoxifen is more effective than either compound alone in halting the growth of MCF7 parental cells (Fig 2C). Metformin may, therefore, have additional functions that are independent of the ERα effects mediated by Tamoxifen.

### Metformin decreases the abundance of MDR markers *in vitro*

**Fig 3:**
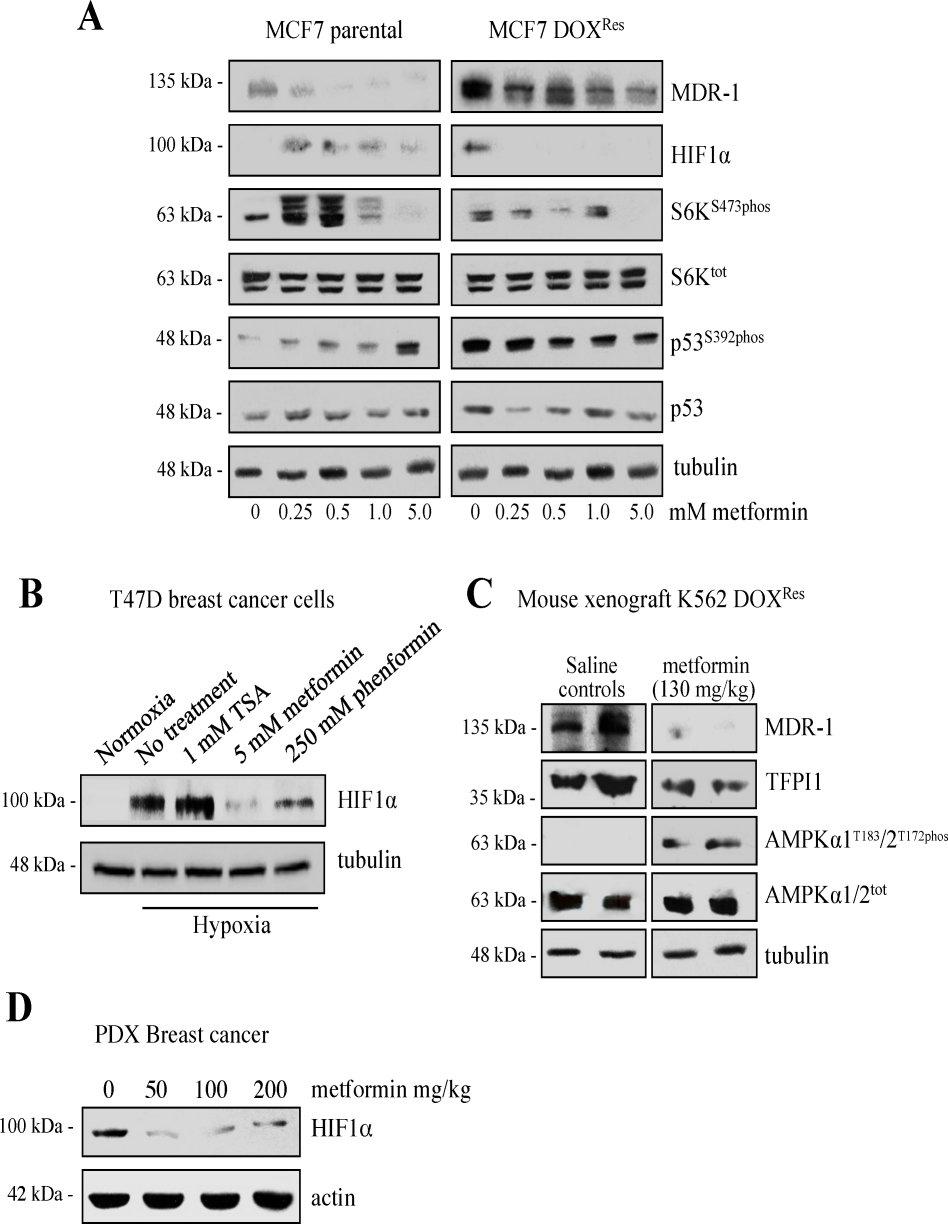
Metformin reverses the expression of cancer related proteins *in vitro* and *in vivo*. **(A)** MCF7 parental and DOX^Res^ cells were treated with a range of metformin concentrations for 48 hours. Equal protein loads of the cell lysates were prepared for Western analyses using the antibodies shown. Results are consistent with three repeat experiments. **(B)** T47D cells were treated with the drugs shown while maintained in a 1% O_2_ hypoxia chamber for 24 hours. Cell lysates were prepared and analyzed using the antibodies shown, representative of two consistent experimental repeats. **(C)** A preliminary study of 2 mice per treatment arm: NOD/SCID mice harboring palpable tumors derived from K562 DOX^Res^ cells were treated with metformin i.p. (130 mg/kg) or saline (considered Day 1). Tumors were excised after 28 days and processed for Westerns using the antibodies shown. The same protein concentration of each lysates was used for all western analysis, with tubulin representing a protein loading control for all. **(D)** A preliminary study of acute metformin effects on tumor markers *in vivo*: NOD/SCID mice growing tumors derived from a human patient with triple negative breast cancer were treated with single specific doses of metformin by i.p (0, 50, 100 and 200 mg/kg body weight) as indicated. 72 hours later, the tumors were excised from each mouse, protein lysates were prepared, equal protein amounts separated by SDS PAGE and assessed by Western analysis using the antibodies indicated. Actin represents the protein loading control. PDX: patient-derived xenograft.

We previously reported that troglitazone (TRG), an insulin sensitizer of the thiazolidinedione class used in the treatment of DM2 (metformin is an unrelated biguanide insulin sensitizer), reduced the protein levels of the ABC transporter class of drug efflux pumps elevated in multiple aggressive cancer cells (MDR-1 and BCRP in MCF7 DOX^Res^, and MDR-1 in K562 DOX^Res^ cells) [15]. Additional recent reports by others indicate that metformin may also possess this ability *in vitro* [26, 27]. Considering that metformin reduced the abundance of ERα (Fig 2B), and decreased the proliferation of MCF7 parental and DOX^Res^ cells, we asked if metformin could decrease the abundance of multiple markers of MDR as a means of explaining the enhanced treatment sensitivity of MDR cells upon metformin treatment. We also asked if metformin had effects across drug-resistant cell populations of unrelated cancer types. We first looked *in vitro* at our MCF7 cell line selected for resistance to DOX (Fig 3A). We directly compared the relative protein abundance of MDR markers between parental and MCF7 DOX^Res^ cells across a wide concentration range of metformin. At baseline without metformin (Fig 3A, compare 0 lanes), the DOX^Res^ population displayed enhanced protein abundance of these MDR protein markers compared to sensitive cell populations. In turn, there was a trend to decrease the abundance of MDR-1, HIF1α, p53^S392phos^ (versus total p53) and S6K^S411phos^ (versus total S6K) in a dose-dependent manner that was much more prevalent in the MCF7 DOX^Res^ cell population. We included an assessment of p53 ^phos S392^ since p53^phosS392^ in MCF7 parental cells is a beneficial activating and stabilizing signal that acts to target cancer cells for apoptosis [28], but in aggressive cancer cells, p53^phos392^ is often elevated, with this contributing to tumor progression [29, 30].

We observed several marked differences in the abundance of MDR protein markers between MCF7-sensitive and -resistant cell populations in response to metformin (Fig 3A). First, in parental/sensitive cells, HIF1α protein levels were consistently increased by metformin, even at the lowest concentration tested. Secondly, abundance of phospho-, but not total p53 and S6K were enhanced in parental cells in a dose-dependent manner (Fig 3A, left panels). Next, in resistant populations (MCF7 DOX^Res^), metformin reduced p53^phos392^ levels in a dose-dependent manner, which was also observed for the protein levels of HIF1α, MDR-1 and S6K^S411pho^ (Fig 3A, right panels). Taken together, these results are consistent with metformin activity being context dependent, with metformin playing distinct roles in treatment sensitive versus resistant MCF7 cells.

Metformin exposure decreased HIF1α protein levels under normal oxygenation levels (Fig 3A). Hypoxia is a powerful inducer of HIF1α expression, just as the hypoxia present in the intratumoral environment is considered a major driver of aggressive and treatment resistant tumors [31, 32]. We queried whether metformin was capable of blocking the accumulation of HIF1α under strong hypoxic induction conditions. We observed that hypoxia was sufficient to markedly induce the expression of HIF1α protein (Fig 3B), but that metformin was the most effective agent tested that blocked hypoxia-induced HIF1α protein abundance. Indeed, it was more effective than a second biguanide, phenformin, in inhibiting HIF1α protein expression. Unlike metformin however, phenformin is not in clinical use.

### Metformin dependent reversal of MDR-associated protein markers occurs *in vivo* in breast and non-breast cancers

We next asked if metformin demonstrated similar efficacy in lowering MDR markers in an *in vivo* model. In this preliminary study, we grew human tumors in mice from both leukemia cell lines (K562) and patient-derived (PDX) breast cancer cells. Specifically, we injected severely immunodeficient NOD-SCID-IL-2Rgamma^-/-^ (NSG) mice with K562 human myelogenous leukemia cells selected for DOX^Res^ [16], but also xenografted tumor fragments obtained from a patient-derived TN partially-resistant breast tumor (based on actual clinical response) into NSG mice (4–28). Once tumors formed (~25 mm^3^; day 11 for K562 DOX^Res^ cells, and day 37 for 4–28 tumor slices), metformin was administered by i.p. injection. NSG mice growing K562 DOX^Res^ cells were injected on alternating days (days 11–28) and sacrificed on day 28, whereas the 4–28 tumor-bearing mice were sacrificed 72 hours after a single metformin treatment. The excised tumor tissue was assessed for changes in MDR-1 and TFPI1 protein abundance in response to metformin exposure (we previously found TFPI1 involved in MDR development [14]) (Fig 3C), as well as HIF1α protein abundance (Fig 3D).

Metformin monotherapy did not decrease the size, nor slow the growth of the K562 DOX^Res^ tumors, as compared to the untreated control arm *in vivo* in this pilot test. Despite this lack of clinical response, the MDR-1 and TFPI1 protein levels did noticeably decrease in the metformin treated arms (Fig 3C). Metformin was physiologically active in the NSG mice, based on the ready detection of AMPK phosphorylation in the metformin treatment arm (Fig 3C). In our metformin dose response pilot study using 4–28 human patient-derived tumor fragments, HIF1α levels were reduced even at the lowest metformin concentration (50 mg/kg; Fig 3D). The above results suggest that metformin reduces MDR markers both *in vitro* and *in vivo*, in breast and non-breast cancers.

### Metformin-dependent reversal of MDR markers in resistant cell populations is AMPK-independent

We next investigated whether metformins’ antiproliferative activity is NFκB–or AMPK-dependent, and tested this in both sensitive and resistant cell populations. A previous report suggested that the metformin-dependent decline of MDR-1 protein levels in MDR cells is mediated through inhibition of NFκB activity [26]. However, we observed that NFκB is reduced in MCF7 DOX^Res^ cells, and that metformin did not appreciably impact NFκB protein levels in the parental cell population in a dose dependent manner (S1 Fig). Consistent with this, we found that silencing NFκB did not impact metformin antiproliferative activity (S2 and S3 Figs). We conclude that NFκB is not required for the antiproliferative activity of metformin on treatment resistant MCF7 cells.

The metabolic beneficial activity of metformin in DM2 is believed to occur in part via the phosphorylation and activation of AMPK [33]. Studies have shown that metformin facilitates the phosphorylation of AMPK indirectly through the action of the upstream kinase LKB1 [34,35]. In contrast to the known contribution of AMPK/LKB1 in metformin’s glucoregulatory activities, it remains unclear how metformins’ antiproliferative activity is regulated, and is still being debated [36–38]. A recent report indicated that metformin required AMPK, but not LKB1, to halt the proliferation of human non-small cell lung cancer cells [37].

**Fig 4:**
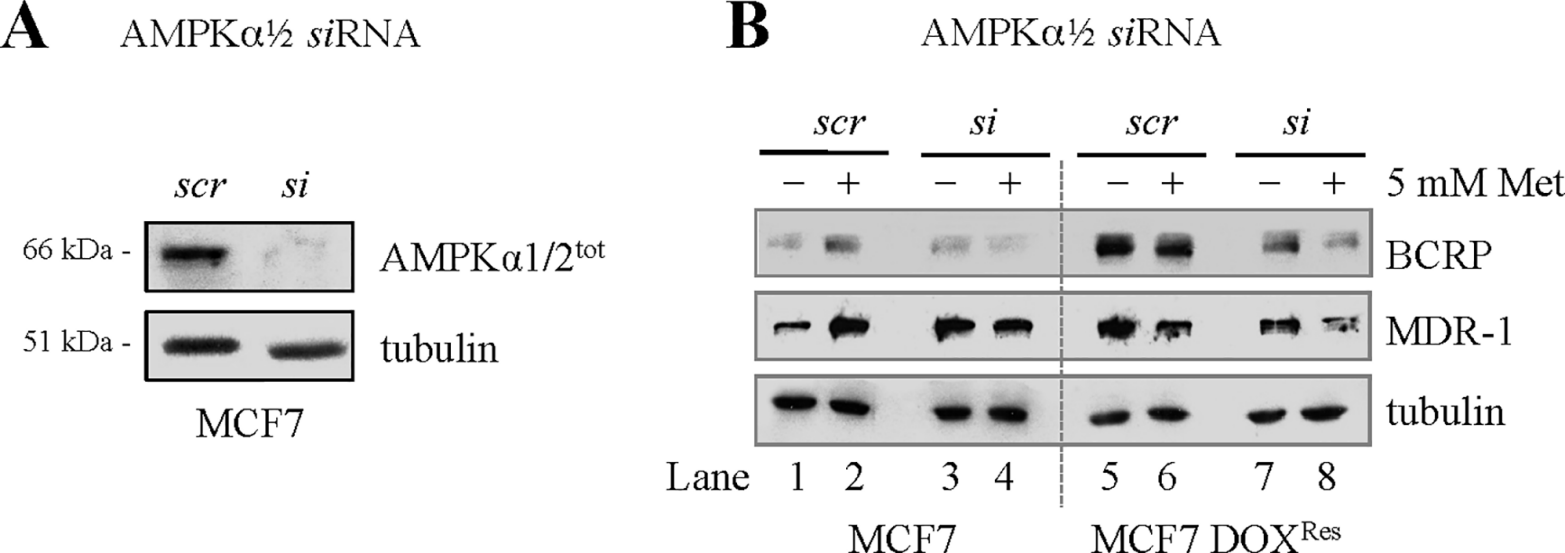
AMPKα1/2 is not required for metformin activity on cancer protein abundance. **(A)** Scrambled (*scr*) and targeted siRNAs (*si*) against AMPKα1/2 were transfected into MCF7 parental cells. Lysates were prepared and Westerns were performed using the antibodies shown. Immunoblot results were consistent over three biological repeat experiments. **(B)** AMPKα1/2 was silenced in MCF7 parental and DOX^Res^ cells, followed by metformin addition. Controls were left untreated. Cell lysates were prepared and assessed using the antibodies shown: the immunoblot represents consistent results observed for three biological repeats.

To test the AMPK-dependence of metformin-induced decreases in MDR-associated proteins in breast cancer, we silenced AMPK in treatment sensitive and resistant MCF7 cell populations (Fig 4). As noted previously, there was an increase of MDR-1 and BCRP markers in the treatment resistant cell population, in the absence of treatment (Fig 4B, lane 1 vs. 5). In the resistant cell population, metformin tended to decrease both MDR-1 and BCRP protein levels regardless of AMPK silencing (Fig 4B, Lane 5 & 6 vs. Lane 7 & 8). In contrast, metformin treatment in parental MCF7 cells caused MDR-1 and BCRP levels to increase in controls cells (scrambled *si*RNA; *scr*), which was partially reversed in AMPK-silenced cells (Fig 4B, left panel). One interpretation of this is to suggest that in DOX^Res^ cells, metformin acts in an AMPK-*independent* manner. This suggests that MDR-1 and BCRP, as members of a stress response system [39] are activated in the presence of metformin, and this appears to be AMPK-*dependent*. This supports the possibility that metformin uses different cellular machinery depending on sensitive versus drug resistant cell populations.

### Metformin possesses indirect HDACi activity

**Fig 5:**
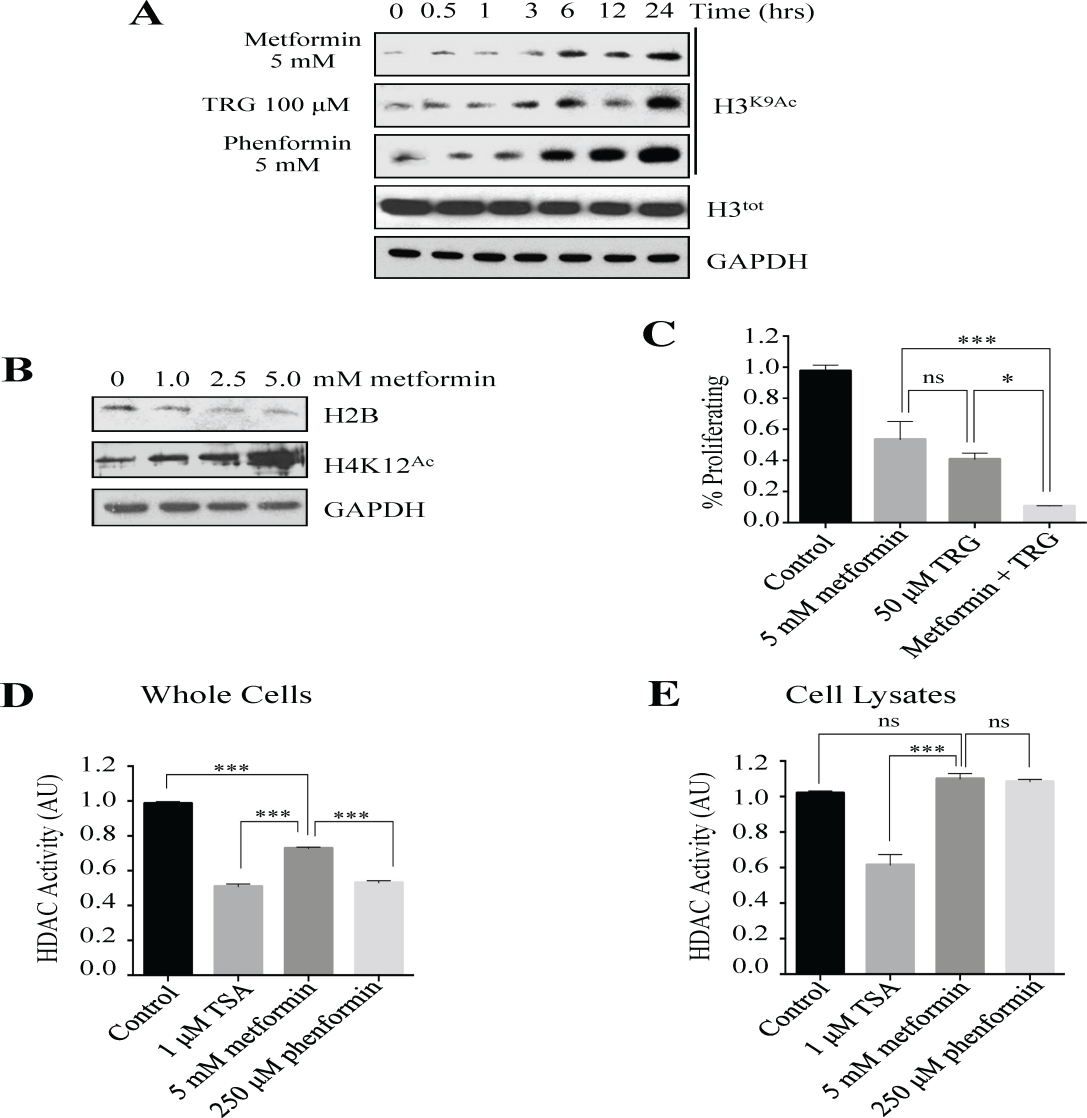
Metformin possesses indirect HDACi activity. **(A)** MCF7 parental cells were treated with metformin (5 mM), TRG (100 μM), or phenformin (5 mM) for 24 hours. Cells were removed at the times shown and lysates assessed by Western analysis using the antibodies indicated. The immunoblot is representative of duplicate biological repeats. **(B)** Western analyses of MCF7 cells following 48-hour exposures to various metformin doses. Primary antibodies are indicated, and represent three consistent repeat experiments. **(C)** MCF7 cells were treated with doses of metformin (5 mM) and/or TRG (50 μM) for 48 hours. Proliferation was measured by MTT assays from four biological repeats. n = 4 samples analyzed **(D)** HDAC assays were performed on lysates obtained from T47D cells treated with the drugs shown for 48 hours. HDAC activity was measured on three biological repeats, and reported as % Activity using arbitrary units (AU) normalized to control. n = 6 samples analyzed. **(E)** 80 μg of protein prepared from parental T47D cells was treated with the indicated concentrations of TSA, metformin or phenformin for 30 minutes at 30°C. HDAC activity was measured in duplicate, on three biological repeats. n = 6 samples analyzed. * = p < 0.05, *** = p < 0.001, ns = not significant.

We had previously observed that the non-biguanide insulin sensitizer Troglitazone (TRG), a thiazolidinedione class of drugs used to treat DM2, increased histone posttranslational modifications (15, 16, 40),. We asked if metformin was also capable of modifying histones, given that this modification may alter gene expression and explain in part the altered MDR-marker protein levels we have seen with metformin exposure. We performed Western analyses of histone H3 Lys9 acetylation (H3K9^Ac^) in MCF7 cells with increasing durations of metformin, TRG and a second biguanide, phenformin, and observed that a fixed dose of metformin tended to increase H3K9^Ac^ in a time dependent manner (Fig 5A) and that metformin and phenformin both exhibited a comparable temporal induction of histone acetylation to that of TRG. Metformin also induced H4 acetylation (H4K12^Ac^) in a dose dependent manner (Fig 5B), while total histone H2B remained unchanged with time and dose (Fig 5A and 5B). This may be due to an underlying histone deacetylase inhibitor (HDACi) activity.

Although from unrelated drug classes, we found the insulin sensitizers TRG and metformin to induce global histone acetylation, and to exert cytotoxic effects on MCF7 cells. To determine if they are acting through separate or similar pathways, we compared the cytotoxic effects of the drugs when used alone or in combination. The results show that cell proliferation is most greatly impaired when both metformin and TRG are used in combination (Fig 5C), supporting the idea that separate pathways are utilized by TRG and metformin when it comes to inhibiting cancer cell proliferation.

We had previously provided evidence that increased global histone acetylation in response to TRG exposure was due to an inherent and direct HDACi activity [40]. This is highly relevant, as HDACi’s possess antiproliferative activity in cancer cells and are presently in phase III clinical trials [41,42]. To test whether the increases in histone acetylation noted here with metformin are also due to HDACi activity, we performed *in vitro* quantitative assays to measure the deacetylation of an acetylated peptide, as done previously [40]. To test for the presence of indirect histone deacetylation activity, we measured HDAC activity in whole cell lysates collected after the cells were exposed to the classic HDACi Trichostatin A (TSA), as well as the biguanides metformin and phenformin. The lysates were incubated with acetylated peptides that fluoresce when deacetylated, allowing quantitative measurement. Compared to untreated controls, all chemical agents significantly inhibited HDAC activity (Fig 5D). Since this could occur via a cell signaling mechanism within whole cells (indirectly) and not by direct inhibition of HDAC enzymes, we repeated the assay in untreated cell lysates to determine if there is indirect HDACi activity. TSA again blocked the deacetylation of the peptide, whereas metformin and phenformin did not (Fig 5E). Together, these results are consistent with the biguanide compounds, metformin and phenformin, indirectly inhibiting HDAC activity, rather than by directly affecting HDAC enzymes.

### The development of treatment resistant breast cancer can be prevented by pretreatment with metformin *in vitro*

**Fig 6:**
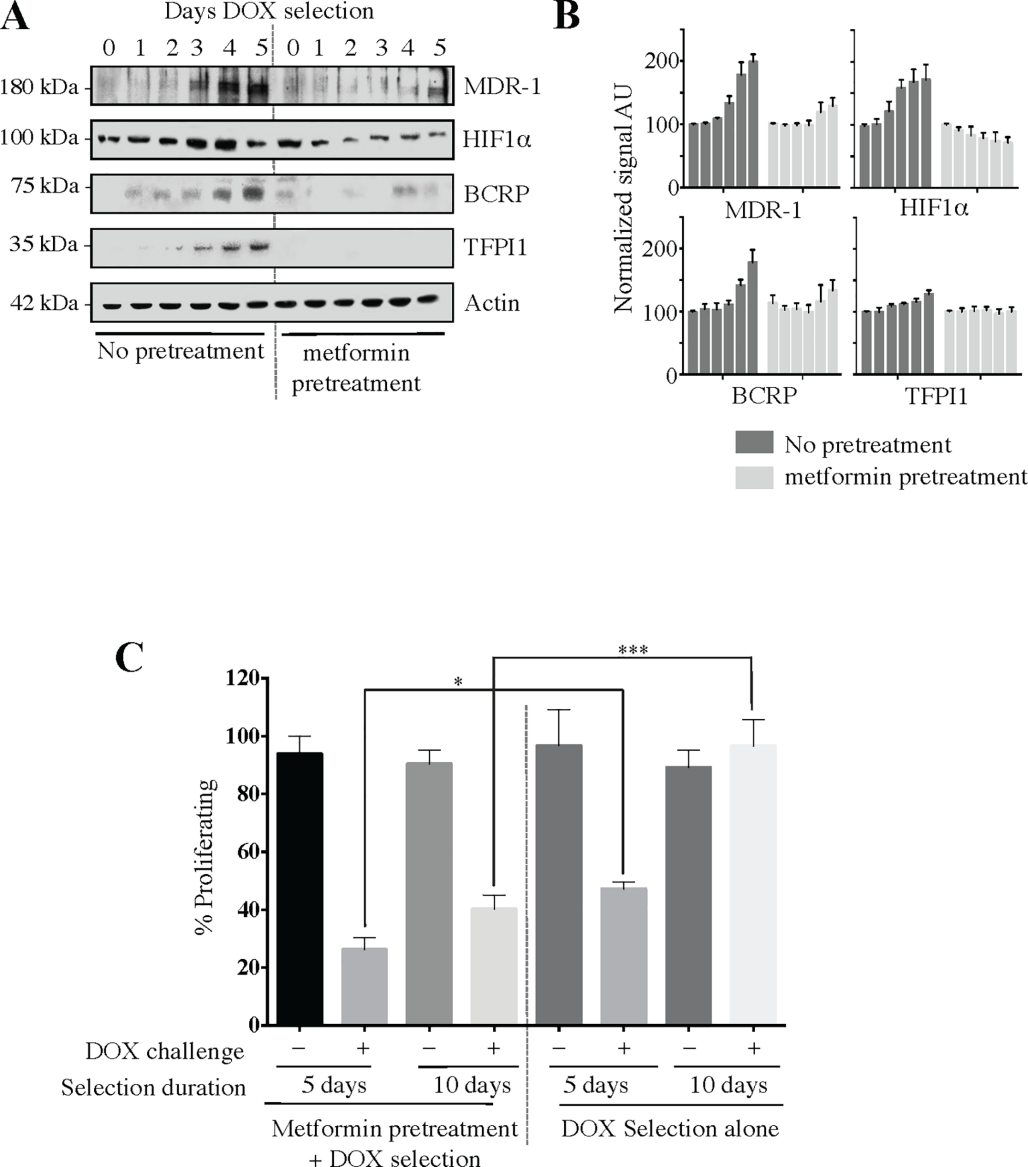
Pretreatment of cells with metformin prevents acquired DOX resistance and markers of MDR. **(A)** MCF7 cells were selected for DOX resistance according to our standard methods (1 μM DOX for 48 hours, followed by maintenance of cells in 100 nM DOX for up to 2 weeks), in triplicate, with one set of cells pre-exposed to metformin (pretreatment; 100 μM) before and during DOX selection. Samples were removed every 24 hours for 5 days for Western analyses. Immunoblot is consistent with results for all repeats. (B) Semiquantitation of individual protein target Western blot signals from triplicate experiments represented in (A), highlighting the differences per Day of DOX selection between no pretreatment versus metformin pretreatment. Values were normalized to actin signal, and Day 0 set at arbitrary value of 100. **(C)** Trypan-blue assay of survival of DOX-selected cells exposed to DOX (1 μM, 48 hours) with, and without, metformin pretreatment. Three biological repeats were done in duplicate, with testing on Day 5 and 10 of selection. n = 6 samples analyzed. * = p < 0.05, *** = p < 0.001.

We asked whether the metformin-dependent reductions in MDR marker protein levels in resistant populations could be adapted for use in the sensitive parental populations to prevent the development of resistance to therapy. To test this, we used standard selection pressure in MCF7 cells with, or without, the presence of metformin pretreatment to compare the resulting cell population’s sensitivity to a sublethal dose of DOX. Specifically, the parental MCF7 cells were pre-treated with a low concentration of metformin (100 μM) for 48 hours, which was continued throughout the experiment, in the presence of DOX selection pressure. Cell lysates were prepared from the treated and untreated cells every 24 hours and analyzed for MDR protein marker expression (Fig 6A). Metformin pretreatment delayed/decreased (HIF1α, MDR-1, and BCRP) or prevented (TFPI1) the protein increases typically observed in DOX^Res^ cells (compare right side with left side in Fig 6A and 6B). These results demonstrate that pretreatment of cells with low dose metformin effectively inhibits the expression of MDR-associated proteins.

Of importance when considering future clinical implications of our findings, we next compared the drug sensitivity between these cell populations selected for resistance while in the presence of metformin. On days 5 and 10 during the DOX selection process, cells were exposed to 1 μM DOX to test for resistance to this drug. The results demonstrate that metformin pretreated cells retained DOX sensitivity as compared to those without metformin pretreatment at Day 5 and even greater differences at Day 10 (Fig 6C). There appears to be a correlation between the metformin-dependent decreases in MDR protein markers and maintenance of treatment sensitivity despite the strong drug selection protocol. However, in cells without metformin pretreatment, by Day 10 there was no significant DOX treatment effect on proliferation, suggesting that drug-resistance was developing over the experimental timeline. Interestingly, even though markers of MDR were elevated on Day 5 (Fig 6A and 6B), the cells remain drug sensitive, indicating that it may be possible to detect early development of MDR prior to clinical relevance. This observation provides evidence that metformin, at a low and potentially clinically relevant dose, has the capacity *in vitro* to block the development of drug resistance, and that elevated MDR markers may precede drug resistance.

One cellular proliferation assay we utilized was the MTT assay [3-(4,5-dimethylthiazol-2-yl)-2,5-diphenyltetrazolium bromide] to measure the cytotoxic/static effect of metformin exposure in drug resistant breast (MCF7) and leukemia (K562) cell lines. Yet we also utilized two independent measures of proliferation, that of thymidine incorporation (TI) and trypan blue exclusion, to directly compare the measurements obtained by MTT, as there is a theoretical risk that metformin may influence MTT results by falsely lowering the measure of proliferation due to mitochondrial effects. MTT is reduced to a purple formazan in living cells by NAD(P)H-dependent oxidoreductase (NOX) enzymes [43]; NOX enzymes produce reactive oxygen species (ROS) in the cytosol, which may be inhibited by metformin [44], thus falsely lowering the purple chromophore indicating live cells, and therefore appear as an underestimate of cell viability. In contrast, TI measures replicating DNA, and would not be similarly affected by metformin. As shown in S4 Fig, the proliferation measured in the presence of 1 mM and 5 mM metformin is not significantly different between the two assays, supporting our use of the MTT assay in the presence of these concentrations of metformin. Next, we used Trypan blue exclusion assays (S5 Fig); trypan blue is a dye that is excluded from living cells, presumably due to an intact and impermeable plasma membrane, and is considered a measure of viability, not just proliferation [45]. S5 Fig indicates that the MTT assay on MCF7 cells treated with increasing doses of metformin up to 5 mM does not underestimate cell viability/proliferation. Our comparison of both TI and trypan blue exclusion assays to those values obtained by MTT assays indicated that metformin had no discernable impact on our proliferation assessments, and that MTT assays can provide valid measures for these experiments.

## Discussion

### Adjunct metformin resensitizes established MDR to treatment *in vitro*

We have demonstrated that metformin monotherapy has an antiproliferative effect on multiple cell lines, including those selected for resistance to Doxorubicin (DOX) (Fig 1 and Fig 2), in a dose dependent manner (Fig 1A). This extends to multiple ERα^+^ and ERα^-^ breast cancer cell lines (Fig 2A). The effect is also observed when metformin is used in combination with other anticancer treatments in breast cancer cells (Fig 1B and Fig 2C). Our findings are consistent with the growing literature base demonstrating metformin’s ability to slow the growth of tumor cells *in vitro* [46]. Furthermore, our results provide a rationale for the clinical use of metformin in ERα^-^ and more aggressive breast cancers, which are inherently more difficult to treat [47].

### Metformin reverses the levels of MDR-associated protein markers

We have shown that metformin can reverse the expression of MDR markers *in vitro* and *in vivo* (Fig 3 and Fig 4B), and this appears to be linked to the ability of metformin to render DOX^Res^ cells sensitive to DOX once again (Fig 1B). Metformin reduced the expression of MDR markers *in vitro* and also in 2 separate pilot studies using *in vivo* mouse models of aggressive tumors, one derived from a K562 human leukemia cell line selected for resistance to DOX (Fig 3C), and one from a tumor obtained from a triple negative breast cancer patient (Fig 3D). We observed that metformin acted on multiple cancer-promoting pathways in DOX^Res^ MCF7 cells by reducing the expression of at least two members of the ABC drug antiporters, MDR-1 and BCRP, reducing cancer-related phosphorylation of S6K on serine 473, and p53 on serine 392 [29], and by reducing the expression of HIF1α (Fig 3A and 3D). We previously reported that the anticoagulation cascade plays a role in MDR development: the coagulation Factor Xa inhibitor Tissue Factor Pathway Inhibitor 1 α (TFPI1α) is strongly upregulated in breast cancers *in vitro* and within patient samples upon MDR selection [14]. Metformin is also capable of reducing TFPI1α protein levels in a mouse model of treatment-resistant human leukemia (K562 DOX^Res^) (Fig 3C). Thus, metformin is capable of inhibiting multiple cancer promoting pathways in cells.

While DOX is commonly used as a chemotherapy agent to treat many cancers, it has limitations as it induces cardiomyopathy, resulting from mitochondrial dysfunction and the generation of reactive oxygen species (ROS) [48]. DOX inhibits mitochondrial complex I activity, leading to reduced ATP generation and loss of membrane potential. This results in release of cytochrome c from mitochondria, followed by the activation of caspase 3, DNA fragmentation and finally, apoptosis. The effects of metformin on MDR cells, according to our studies (reduction of drug efflux pumps), potentially creates a situation where DOX may no longer be actively pumped out of the cell, allowing DOX to have a more potent effect once again. Opposed to this interaction, metformin has been found to relieve DOX induced cardiomyopathy in cardiomyocytes when cells were pretreated with metformin prior to DOX addition [48]. Metformin is an energy disrupter that also targets and inhibits complex I within the mitochondrial inner membrane [49]. Thus, it seems unlikely that metformin would rescue mitochondrial dysfunction in DOX treated cells (which also inhibits complex I). However, recent studies indicate that metformin also induces ER stress that causes the release of calcium from the ER that is taken up by mitochondria, leading to mitochondrial swelling and mitochondrial biogenesis [50]. This was proposed to explain why metformin has weak apoptotic ability in cancer cells, but also provides a likely explanation for how metformin could rescue mitochondrial defects in DOX treated cardiomyocytes.

Metformin has been described as impacting a broad array of molecular mechanisms, and may influence different mechanisms depending on the chemotherapeutic drug it is combined with [51,52]. Metformins’ ability to “poison” mitochondria was also shown to be relevant to reversing resistance to Tamoxifen in MCF7 cells. It was concluded that mitochondrial activity was crucial to the development of Tamoxifen resistance, and use of metformin to block mitochondrial function was enough to reverse the resistant phenotype [53]. Others have likewise shown that metformin also has an anti-cancer effect on Tamoxifen resistant cells *in vitro*, and that this occurs in ER positive cells [54,55]. This is consistent with our observations that metformin has an increased toxic effect on ER^+^ cells compared to ER^-^ cells, that metformin reduces ER expression, and that metformin works synergistically with Tamoxifen (Fig 2C)

Metformins’ ability to reduce the expression of markers of aggressive cancer was not limited to drug resistant cell populations, as metformin also prevented the expression of HIF1α following exposure to hypoxia (Fig 3B). We did not investigate whether this was through a decrease in *HIF1α* transcription versus through enhanced degradation [56], although *HIF1α* transcription is normally constitutive regardless of oxygen abundance, whereas multiple post-translational modifications impact HIF1α protein stability in response to oxygen levels [57]. The observed effect of metformin on HIF1α is important, as many tumor microenvironments are hypoxic, providing the stimulus for HIF1α protein stabilization, which is associated with increased angiogenesis and cell survival [58–60]. Consistent with this, we previously reported that cells that survive hypoxia are resistant to a subsequent exposure to DOX [14]. It is clear in the literature that hypoxia plays a vital role in the development of drug resistance [61–63], thus the ability of metformin to block the expression the HIF1α protein upon hypoxic induction is highly relevant to its ability to prevent/reverse drug resistance.

As a means of understanding the mechanism involved in blocking HIF1α gene expression under hypoxic conditions, we found that Trichostatin A (TSA), a direct HDACi with potent antiproliferative activity, did not diminish HIF1α protein levels following hypoxia, in contrast to the decreases observed with metformin exposure (Fig 3B). We had shown that TRG reduced the accumulation of MDR-1 and BCRP in MCF7 DOX^Res^ cells, similar to metformin, but had not included HIF1α in those studies [15]. A previous report suggested that TRG might in fact induce HIF1α mRNA and protein levels [64] so it remains unclear why metformin and TRG differ in this HIF1α hypoxic effect. Both metformin and TRG are insulin sensitizers and both inhibit HDAC activity (Fig 5D and 5E; [40]). However, TRG appears to have direct HDACi activities, possibly by binding HDAC enzymes, whereas metformin seems to have an indirect HDACi effect, likely by interfering with signaling pathways that activate HDAC activity. Thus, it appears that the hypoxic inhibition of HIF1α by metformin may be independent of its HDACi activity.

### Metformin acts independently of AMPK and NFκB in MDR cells

Metformin facilitates optimal glucose control in Type 2 diabetes (DM2) at least in part by targeting AMPK for activation [35], which in turn affects key players in catabolic metabolism and glucose uptake pathways [65]. However, we show that metformins’ action within drug-resistant cell populations can occur in an AMPK-independent way, as MDR-1 and BCRP protein levels were still reduced by metformin following AMPK silencing (Fig 4). It has also been suggested that the antiproliferative ability attributed to metformin exposure lies in its ability to downregulate NFκB activity, in an AMPK-dependent manner [26]. We found that NFκB protein levels were dramatically reduced in MCF7 DOX^Res^ cells, compared to MCF7 parental cells, where metformin had little effect on NFκB protein expression (S1 Fig). Furthermore, silencing NFκB mRNA expression in MCF7 parental cells had little effect on metformin cytotoxicity (S2 Fig and S3 Fig). Thus, neither AMPK nor NFκB appear required for metformin action, in this cell line, and under these conditions.

### Metformin exhibits multiple anticancer activities

The observed decreases in ABC transporters, HIF1α, and TFPIα protein levels, and the enhanced phosphorylation of S6K^S473^ and p53^S392^ clearly define a variety of potential pathways that metformin may influence in order to control cancer cell proliferation. However, the underlying mechanisms clearly remain elusive and involve AMPK-dependent and AMPK-independent mechanisms [66]. We reported previously that the chemically unrelated thiazolidinedione class (TRG) of insulin sensitizers induced increased histone acetylation via direct HDACi activity [40]. TRG also decreased the phosphorylation of S6K and AKT, similar to that observed with metformin (Fig 1D and Fig 3A; [40]). Similar to TRG, metformin induced a dose-dependent increase in histone acetylation (Fig 5A and 5B), yet this appears to be an indirect effect (Fig 5E) that is consistent within the biguanide class of drugs, as phenformin acted similarly in this assay (Fig 5D and 5E). A direct HDACi activity suggests that the drug can directly bind to an HDAC enzyme and block its function, whereas an indirect activity indicates that a signaling pathway is activated that in turn may block HDAC activity. There is a possible explanation for the indirect metformin HDACi effect, as metformin has been shown to cause the LKB1- and AMPK-dependent phosphorylation of class IIa HDACs (HDAC4, 5 and 7), which leads to their nuclear export and loss of targeted activity [67].

Histone acetylation is an important determinant of gene expression, with increases associated with elevated transcription, whereas deacetylated histones are often associated with gene repression [68]. HDACi’s are potent killers of cancer cells [69], and several HDACi-based cancer therapeutic strategies are currently in clinical trials [70]. We found that metformin likely acts through a different mechanism than TRG since treatment of MCF7 cells with both TRG and metformin caused greater killing than either drug alone (Fig 5C). This synergy was also noted with the combination of metformin and the classic HDACi, TSA [71]. Future work will require establishing the role of metformin in the repression, rather than the expression, of genes and protein modifications critical for MDR development. Metformin may therefore have a clinical niche and usefulness because of its minimal side effect profile, low cost, oral delivery and few contraindications.

### Metformin pretreatment prevents acquired MDR development

The primary goals of this study were to determine whether metformin could reverse and/or prevent MDR. We have established that metformin has the potential to reverse both the MDR-related protein markers and treatment resistance associated with MDR. Next, we asked whether metformin could prevent the onset of MDR, an investigation that would be very difficult using clinical trials, considering the years between initial treatment response and delayed recrudescence. In addition, MDR cannot be detected in most clinical cases until response failure is apparent. Here, we adapted our *in vitro* selection protocol by first pretreating cells with metformin, with a continued presence of metformin during the selection process. By analogy, the daily long-term use of metformin in DM2 has been correlated with the primary prevention of multiple cancer types [72] and improved outcomes when malignancies are present [10–12]. Using low doses of metformin before, and during, the selection process for treatment-resistant cell populations, we observed that this prevented or delayed the accumulation of multiple MDR markers (MDR-1, BCRP and TFPI1), reduced the expression of HIF1α (Fig 6A), and retained a high degree of drug sensitivity. In contrast, rapid accumulation of MDR-protein markers and drug resistance was detected in the absence of metformin pretreatment (Fig 6A–6C). The correlation between lowered protein levels and decreased resistance supports the potential use of these proteins as biomarkers. Future work will extend these timelines to that of established resistant states (after months of selection) to determine if these proteins are transiently or permanently suppressed. Lastly, this may be the first indication of a clinical role for metformin in the long-term management of cancer, where individuals may be maintained on oral metformin to extend remission times, or prevent drug resistance from developing.

## Supporting information

S1 Dataset Minimal manuscript dataset providing the mean, standard error (se), p-values and Bonferroni values. (PDF)

S1 Fig Metformin effects on NFκB protein abundance in MCF7 cell populations. Western analysis of NFκB protein abundance in MCF7 parental and DOX^Res^ cells exposed to increasing concentrations of metformin. The immunoblot is representative of three biological repeats.
(TIFF)

S2 Fig NFκB protein abundance in MCF7 cells upon silencing. Silencing of NFκB was accomplished by transfecting MCF7 cells with *si*RNAs against the NFκB subunit NREL, followed by metformin exposure (+) or not (-). Westerns were performed using antibodies against NREL and NFκB and are representative of three biological repeats.
(TIFF)

S3 Fig Proliferation of MCF7 cells treated with metformin upon NFκB silencing. Proliferation of the cells used in **(S2)** after metformin exposure, and with or without NFκB silencing, did not reveal a significant difference in cellular proliferation, as measured by MTT assays. Performed in triplicate on two biological repeats.
(TIFF)

S4 Fig Comparison of MCF7 proliferation upon metformin exposure using MTT versus thymidine incorporation assays. % Cell proliferation under identical conditions was compared between MTT and thymidine incorporation assays in the presence and absence of metformin (0 mM, 1 mM and 5 mM, 48 hours) in MCF7 cells. n = four biological repeats analyzed. MTT did not underestimate % proliferation in the presence of metformin, with the one-way ANOVA test finding no significant (ns) difference between these two assays; 1mM P = 0.1291; 5 mM P = 0.203.
(TIFF)

S5 Fig Comparison of MCF7 proliferation upon metformin exposure using MTT versus trypan blue assays. % proliferation in MCF7 parental cells using MTT and trypan blue exclusion proliferation/viability assays with increasing doses of metformin (0, 0.1, 1.0 and 5 mM, 48 hours). Three biological repeats, each done in duplicate, did not demonstrate an underestimation of % proliferation in the presence of various metformin doses. One-way ANOVA did not detect a significant difference between MTT versus trypan blue assays for any given metformin dose; 0.1 mM P = 1.00; 1.0 mM P = 0.442; 5.0 mM P = 1.00.
(TIFF)
