# Supplementary material for: Metformin inhibits the development, and promotes the resensitization, of treatment-resistant breast cancer
Source: PLoS One. 2017 Dec 6;12(12):e0187191. doi: 10.1371/journal.pone.0187191 (PMC5718420; doi:10.1371/journal.pone.0187191)
Supplement: S1 Dataset — (PDF) [file pone.0187191.s001.pdf]

Fig 1A

group_1a	mean	se(mean)
Met0	100.6667	1.763834
Met0_dox	66	3.05505
Met1	56.33333	1.855921
Met1_dox	49	2.645751
Met2.5	100.6667	7.446103
Met2.5_dox	91.66667	2.027588
Met5	78.66667	2.905933
Met5_dox	73.33333	2.403701
Total	77.04167	3.954212

Overall ANOVA
F-stat: 31.73
P-value: < 0.00005

Bonferroni

Condition	MFC7-MFC7DOX	p-value
CON	0	1
1.0mM Met	-34.67	<0.0005
2.5mM Met	-35.33	<0.0005
5.0mM Met	-24.33	0.004

Fig 1B

group_1b	mean	se(mean)
Con	92.50936	4.220759
Con_dr	94.70199	4.046423
dox1	61.58968	4.599691
dox1_dr	94.92273	4.561582
met1	54.22389	5.611193
met1_dox	81.89845	3.674021
met1dox	30.91969	2.949362
met1dox_dr	55.40839	4.211652
Total	70.77177	4.794564

Overall ANOVA
F-stat: 30.47
P-value: <0.00005

Bonferroni

Condition	MFC7-MFC7DOX	p-value
CON	-2.19	1
1um DOX	-33.33	0.001
1mM Met	-27.67	0.009
Met + DOX	-24.49	0.027

Fig 2A

group_2a	mean	se(mean)
bt20_met0	83.48	1.539394
231_met0	84.66667	1.466667
t47d_met0	83.83333	6.319371
mcf7_met0	90.93333	7.431764
bt20_met5	42.33333	2.899042
231_met5	44.16667	0.5238745
t47d_met5	15.03333	0.7535103
mcf7_met5	17.66667	2.875954
Total	57.76417	6.279707

Overall ANOVA
F-stat: 68.47
P-value: < 0.00005

Bonferroni

Condition	Met0-Met5	p-value
BT-20	-41.15	<0.0005
231	-40.5	<0.0005
T47D	-68.8	<0.0005
MCF7	-73.27	<0.0005

Fig 2C

group_2c	mean	se(mean)
con	0.4123333	0.0182239
met1	0.1806667	0.0259572
20tam	0.1226667	0.0206989
met_tam	0.0606667	0.0036667
Total	0.1940833	0.0409015

Overall ANOVA
F-stat: 65.12
P-value: < 0.00005

Bonferroni

Comparison	Diff	P-Value
CON - 1mM Met	0.23	< 0.0005
CON - 20uM TAM	0.29	< 0.0005
CON - Met +TAM	0.35	< 0.0005
1mM Met - 20uM TAM	0.06	0.38
1mM Met - Met +TAM	0.12	0.013
20uM TAM - Met +TAM	0.06	0.301

Fig 5C

group_5c	mean	se(mean)
control	97.63333	2.169741
met5	53.46667	6.644881
trg50	40.76667	2.239296
met_trg	10.6	0.2
Total	54.25455	9.689529

Overall ANOVA
F-stat: 72.2
P-value: < 0.00005

Bonferroni

Comparison	Diff	P-Value
CON – 5mM Met	44.17	0.001
CON – 50uM TRG	56.87	< 0.0005
CON – Met + TRG	87.03	< 0.0005
5mM Met – 50uM TRG	12.7	0.34
5mM Met - Met +TRG	42.87	0.001
50uM TAM - Met +TRG	30.17	0.011

Fig 5D

group_5d	mean	se(mean)
con	0.986	0.0102632
tsa1	0.509	0.0141539
met5	0.7283333	0.0075351
phen250	0.5333333	0.0127845
Total	0.6891667	0.057887

Overall ANOVA
F-stat: 371.15
P-value: < 0.00005

Bonferroni

Comparison	Diff	P-Value
CON – 1uM TSA	0.477	< 0.0005
CON – 5mM Met	0.258	< 0.0005
CON – 250uM Ph	0.453	< 0.0005
1uM TSA – 5mM Met	-0.219	< 0.0005
1uM TSA – 250uM Ph	0.024	1
5mM Met – 250uM Ph	0.195	< 0.0005

Fig 5E

group_5e	mean	se(mean)
con	1.02	0.011547
tsa1	0.6133333	0.0592546
met5	1.1	0.03
phen250	1.083333	0.0133333
Total	0.9541667	0.0617725

Overall ANOVA
F-stat: 44.74
P-value: < 0.00005

Bonferroni

Comparison	Diff	P-Value
CON – 1uM TSA	0.407	< 0.0005
CON – 5mM Met	-0.08	0.83
CON – 250uM Ph	-0.63	1
1uM TSA – 5mM Met	-0.487	< 0.0005
1uM TSA – 250uM Ph	-0.47	< 0.0005
5mM Met – 250uM Ph	0.017	1

Fig 6C

group6c	mean	se(mean)
neg_md_5d	93.79333	3.551156
pos_md_5d	26.16667	2.360292
neg_md_10d	90.41667	1.956428
pos_md_10d	40.16667	2.006766
neg_dox_5d	96.71	7.235954
pos_dox_5d	47.21	1.32729
neg_dox_10c	89.12	2.726793
neg_dox_10c	96.6	4.097194
Total	73.63941	4.727071

Overall ANOVA
F-stat: 76.99
P-value: < 0.00005

Bonferroni

Condition	MetDOX-DOX	P-value
5/-	-2.92	1
5/+	-21.04	0.018
10/-	1.3	1
10/+	-56.43	< 0.0005

S3 Fig

group_s3	mean	se(mean)
scr	83.96667	8.591145
scr_met1	35.9	5.164301
nfkb	86	6.921223
nfkb_met1	38.06667	3.131737
Total	60.98333	7.723624

Overall ANOVA
F-stat: 19.46
P-value: 0.0005

Bonferroni

Comparison	Diff	P-Value
scr-scrmet1	48.07	0.004
scr-nfkb	-2.03	1
scr-nfkbmet1	45.9	0.005
scrmet1-nfkb	-50.1	0.003
scrmet1-nfkb	2.17	1
nfkb-nfkbme	47.93	0.004

S4 Fig

group_s4	mean	se(mean)
thy_met0	97.15	1.535415
thy_met1	34.3	7.169496
thy_met5	30.425	6.442615
mtt_met0	92.50936	4.220759
mtt_met1	54.22389	5.611193
mtt_met5	51.72701	2.913917
Total	59.1848	6.289287

Overall ANOVA
F-stat: 30.45
P-value: 0.0005

Bonferroni

Condition	Thy-MTT	P-value
CON	4.64	1
1mM Met	-19.92	0.291
5mM Met	-21.302	0.203

S5 Fig

group_s5	mean	se(mean)
tryp_met0	91.33333	2.333333
tryp_met01	77.4	2.592939
tryp_met1	44.23333	3.083468
tryp_met5	22.1	2.60832
mtt_met0	100.5333	2.575742
mtt_met01	81.23333	1.888856
mtt_met1	53.16667	2.168205
mtt_met5	24.16667	0.6359595
Total	61.77083	5.923677

Overall ANOVA
F-stat: 166.23
P-value: < 0.00005

Bonferroni

Condition	TBE-MTT	P-value
CON	-9.2	0.374
0.1mM Met	-3.83	1
1mM Met	-8.93	0.442
5mM Met	-2.07	1
