# Supplementary figures and images for: Metformin inhibits the development, and promotes the resensitization, of treatment-resistant breast cancer
Source: PLoS One. 2017 Dec 6;12(12):e0187191. doi: 10.1371/journal.pone.0187191 (PMC5718420; doi:10.1371/journal.pone.0187191)

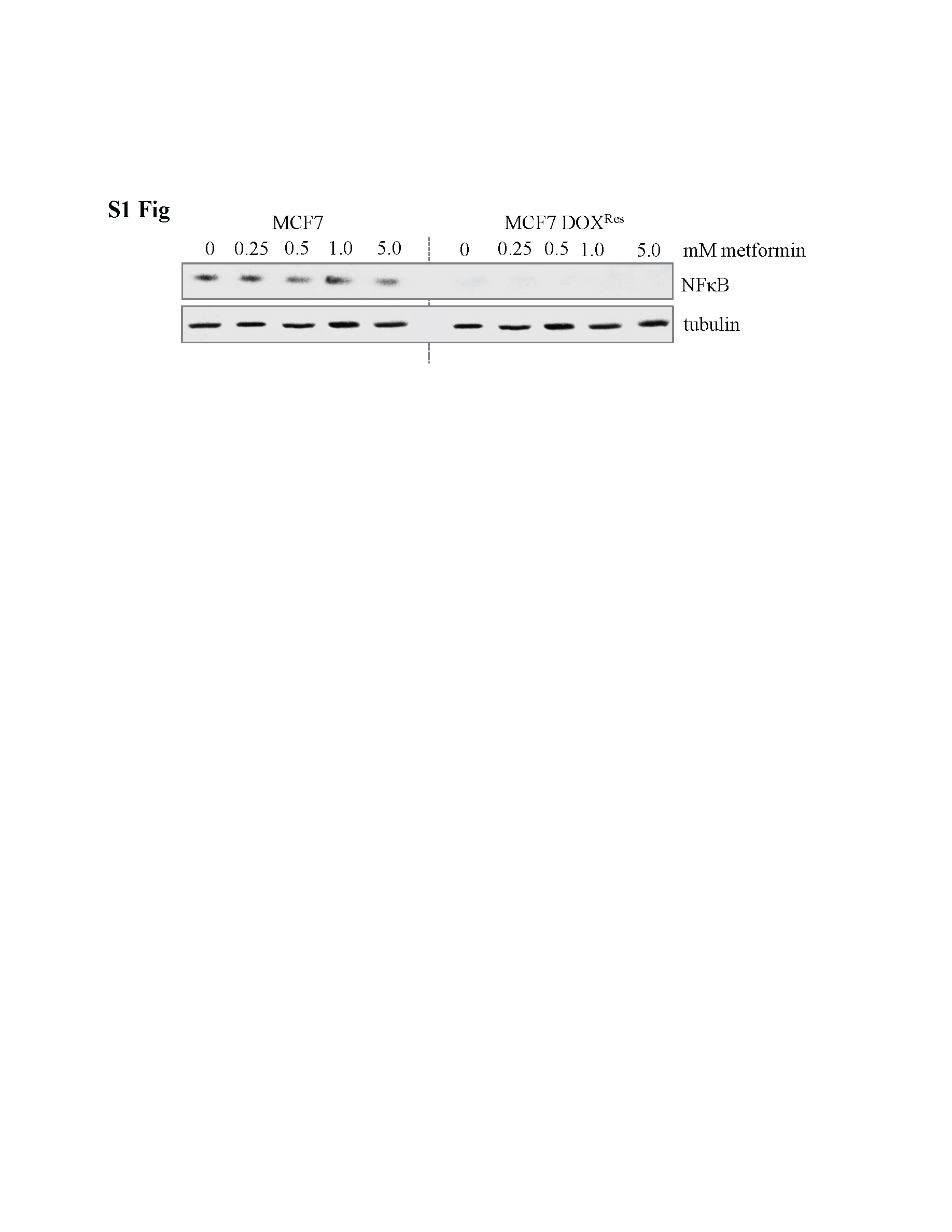

Supplement: S1 Fig — Western analysis of NFκB protein abundance in MCF7 parental and DOXRes cells exposed to increasing concentrations of metformin. The immunoblot is representative of three biological repeats. (TIFF) [file pone.0187191.s002.tiff]

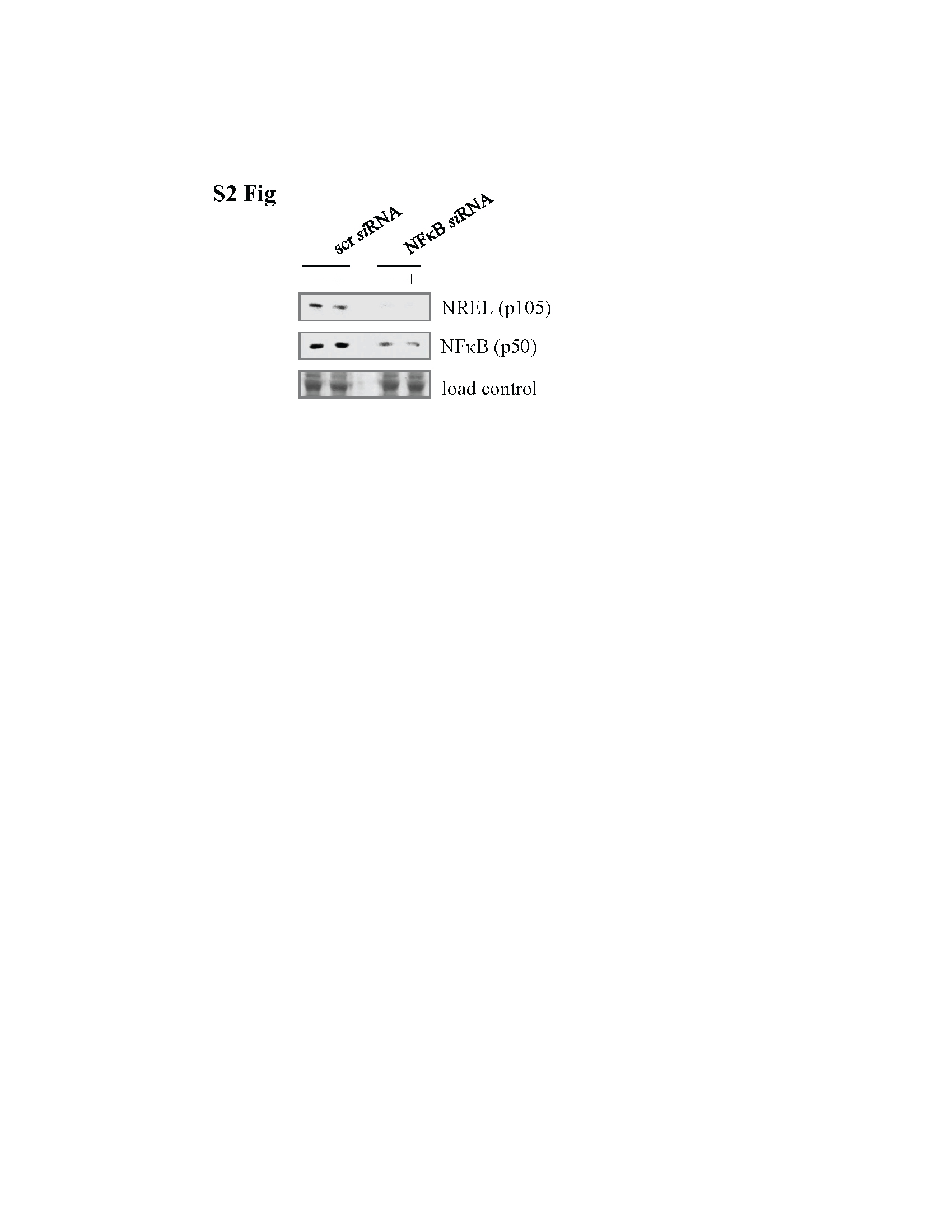

Supplement: S2 Fig — Silencing of NFκB was accomplished by transfecting MCF7 cells with siRNAs against the NFκB subunit NREL, followed by metformin exposure (+) or not (-). Westerns were performed using antibodies against NREL and NFκB and are representative of three biological repeats. (TIFF) [file pone.0187191.s003.tiff]

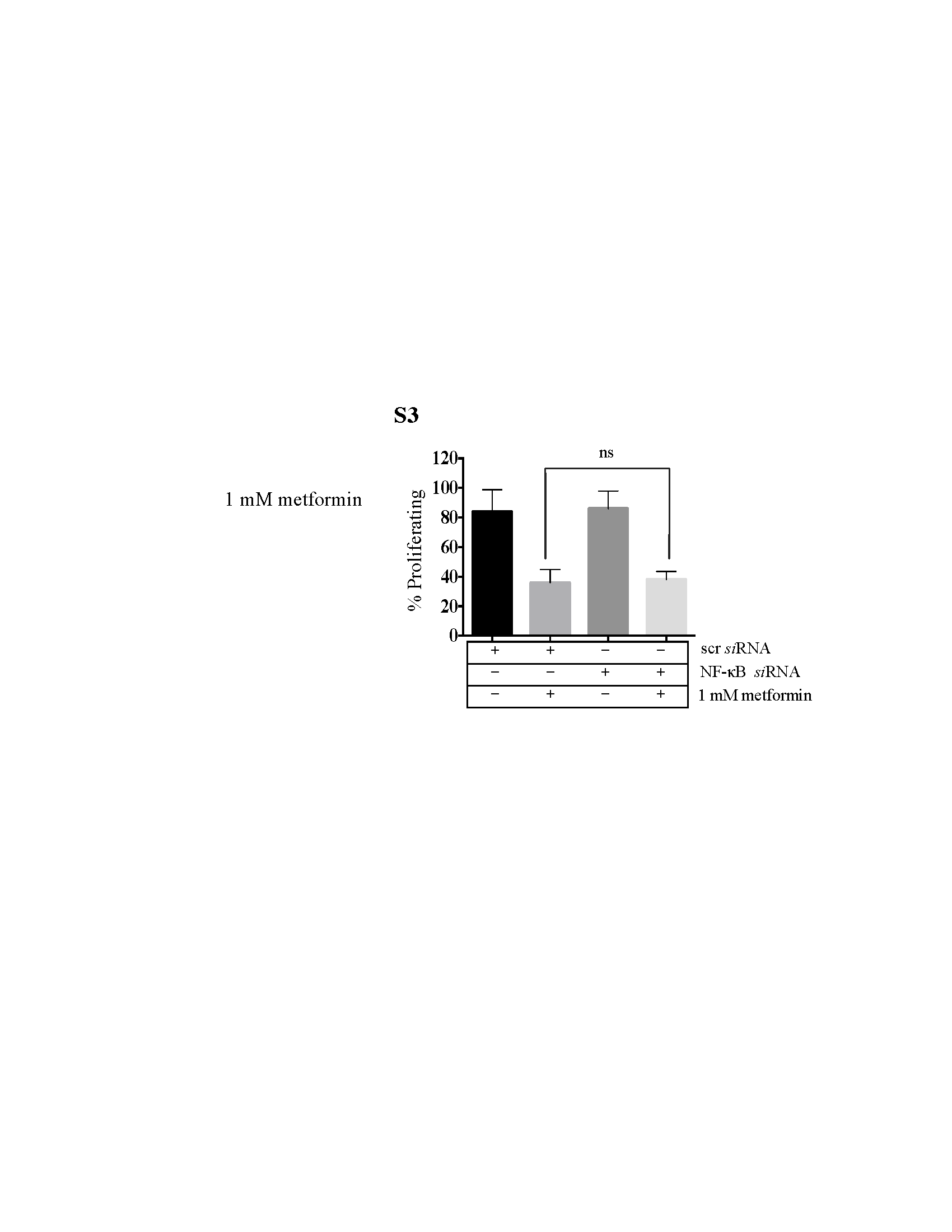

Supplement: S3 Fig — Proliferation of the cells used in (S2) after metformin exposure, and with or without NFκB silencing, did not reveal a significant difference in cellular proliferation, as measured by MTT assays. Performed in triplicate on two biological repeats. (TIFF) [file pone.0187191.s004.tiff]

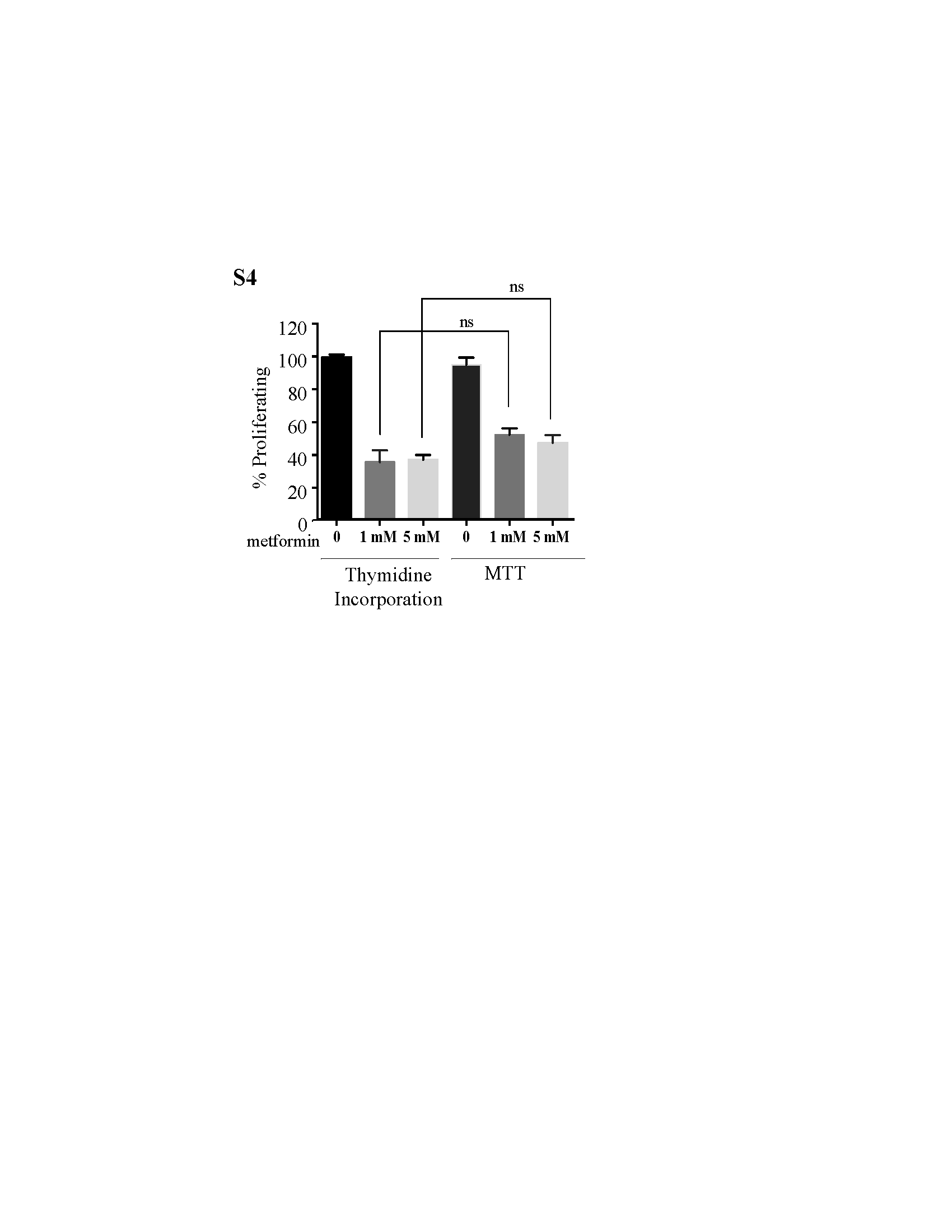

Supplement: S4 Fig — % Cell proliferation under identical conditions was compared between MTT and thymidine incorporation assays in the presence and absence of metformin (0 mM, 1 mM and 5 mM, 48 hours) in MCF7 cells. n = four biological repeats analyzed. MTT did not underestimate % proliferation in the presence of metformin, with the one-way ANOVA test finding no significant (ns) difference between these two assays; 1mM P = 0.1291; 5 mM P = 0.203. (TIFF) [file pone.0187191.s005.tiff]

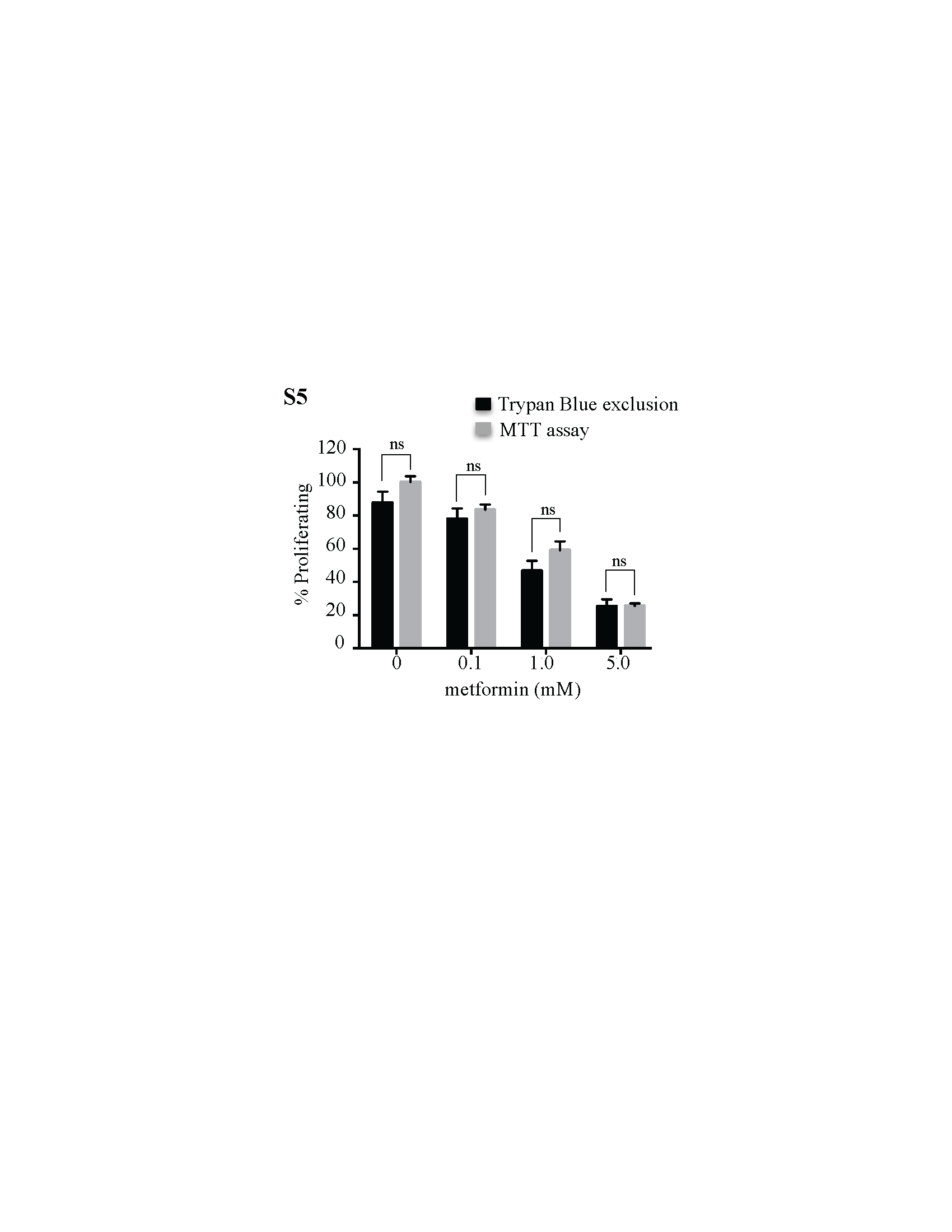

Supplement: S5 Fig — % proliferation in MCF7 parental cells using MTT and trypan blue exclusion proliferation/viability assays with increasing doses of metformin (0, 0.1, 1.0 and 5 mM, 48 hours). Three biological repeats, each done in duplicate, did not demonstrate an underestimation of % proliferation in the presence of various metformin doses. One-way ANOVA did not detect a significant difference between MTT versus trypan blue assays for any given metformin dose; 0.1 mM P = 1.00; 1.0 mM P = 0.442; 5.0 mM P = 1.00. (TIFF) [file pone.0187191.s006.tiff]
